# Causes of ICU readmission and mortality: analysis of a 6-month period

**DOI:** 10.1186/cc12623

**Published:** 2013-06-19

**Authors:** PG Brandão, J Syrio, L Machado, M Guimarães, MV Lopes, SA Lobo

**Affiliations:** 1 Hospital de Base de São José do Rio Preto, São Manoel, São Jose do Rio Preto, SP, Brazil

## Introduction

Patients readmitted to ICU have a higher mortality and longer ICU and hospital stay. Furthermore, the readmission rate is used as a quality indicator of critical care unit performance, because this index may reflect the adequacy of treatment. The objective was to evaluate the readmission rate of a tertiary public hospital during a 6-month period.

## Methods

We performed a retrospective analysis of all adult patients readmitted to a 20-bed mixed-case ICU between 1 September 2012 and 28 February 2013. The cases (readmission) were collected from clinical electronic information system.

## Results

During this period 402 patients were admitted to the ICU. The mortality in the ICU was 24.6% and overall hospital mortality rate was 31.6%. The average SAPS 3 during readmission on the ICU was 52 with a predicted mortality of 34%. The readmission rate was 5.2%, ICU mortality was 23.8% and hospital mortality was 28.6%. The most frequent cause of readmission was nosocomial pneumonia (29%), neurologic causes (19%), sepsis (14%), administrative (14%), postoperative support (10%), metabolic disorders (10%) and cardiology events (5%). The patients were most commonly readmitted form the ward (33%), emergency department (14%), step-down unit (14%), operating theater (5%) and others (33%). The most common supportive therapies after readmission were mechanical ventilation in 38.1%, vasopressors in 28.6%, and renal support in 9.5%.

## Conclusion

The most common reason for ICU readmission in our unit was nosocomial pneumonia. The mortality of the readmitted patients was not superior to the predicted mortality for the overall cohort of patients.

